# Press needle therapy combined with Western medicine for treating acute lower respiratory tract infections caused by respiratory syncytial virus in children under 5 years of age: A retrospective cohort study

**DOI:** 10.1097/MD.0000000000044617

**Published:** 2025-09-19

**Authors:** Juan Meng, Shuqiong Xu, Yanglei Tang, Qingsong Wang, Weijuan Shi, Junru Wang, Lingling Jiang, Danping Huang

**Affiliations:** a Department of Pediatrics, West China Hospital Sichuan University Jintang Hospital, Jintang First People’s Hospital, Chengdu, China; b Department of Traditional Chinese Medicine, West China Hospital Sichuan University Jintang Hospital, Jintang First People’s Hospital, Chengdu, China.

**Keywords:** acute lower respiratory tract infection, children, lung function, press needle, respiratory syncytial virus

## Abstract

The aim of this study was to investigate the clinical efficacy of press needle therapy combined with western medical treatment on acute lower respiratory tract infections of respiratory syncytial virus in children under 5 years old. By retrospectively analyzing the medical records of 83 children hospitalized in the Department of Paediatrics of the First People’s Hospital of Jintang County from September 2024 to April 2025, they were divided into the experimental group (42 cases, treated with press needle therapy combined with western medicine) and the control group (41 cases, treated with western medicine only),observe the symptoms, signs improvement time and airway function of the 2 groups of children after treatment respectively. The results showed that, the time to relieve wheezing after treatment in the experimental group was 2.43 ± 0.51 days, and 2.86 ± 0.46 days in the control group, *P*<.01; the time to relieve shortness of breath in the experimental group was 2.46 ± 0.46 days, and 2.88 ± 0.5 days in the control group, *P*<.01; the time to relieve cough in the experimental group was 5.41 ± 0.58 days, and 5.84 ± 0.70 days in the control group. 5.84 ± 0.70 days, *P*<.05; the time of disappearance of lung signs of children in the experimental group was 5.46 ± 0.52 days, and 5.89 ± 0.52 days in the control group; the VT/kg of children receiving treatment in the experimental group was 8.43 ± 1.37, compared with 7.43 ± 1.50 in the control group, *P* <.05; TPTEF/TE (%) was 28.14 ± 4.16, compared with 24.86 ± 3.09 in the control group, *P* < .01; VPEF/VE (%) was 28.21 ± 4.36, compared with VPEF/VE (%) was 28.21 ± 4.36. The study showed that press needle therapy combined with western medical treatment can effectively improve the symptoms and signs and airway function of children, providing new ideas and methods for clinical treatment.

## 1. Introduction

Respiratory syncytial virus (RSV) is one of the most common pathogens causing respiratory infections in children under 5 years of age worldwide, posing a serious threat to children’s health.^[1]^ It is estimated that approximately 33 million children under 5 years of age are hospitalized annually due to RSV infection, with over 100,000 deaths.^[1]^ Currently, several studies in China have reported clinical and epidemiological characteristics of hospitalized children with RSV infection.^[2–5]^ In 2019, the incidence of acute lower respiratory tract infections (ALRTIs) caused by RSV in children under 5 years of age in China accounted for over 10% of the global total, with hospitalizations representing 17% to 24% of the global total, ranking second globally.^[6]^ The disease burden caused by RSV infection in children is primarily attributed to ALRTIs.^[7]^

Typical RSV infections in children are mainly manifested as lower respiratory tract infection symptoms such as cough, wheezing, and shortness of breath,^[8,9]^ and the western medical treatment is mainly based on symptomatic treatments such as oxygen therapy, antiviral therapy, expectoration, nebulization therapy, and oral medications,^[9]^ and due to the factor of age, the children are not able to cooperate with the treatments according to the expectation, which often leads to unsatisfactory clinical treatment results, prolonged hospitalization, and increased families of the children. This often leads to unsatisfactory clinical results, prolonged hospitalization and increased economic burden for the families of the children. In recent years, integrated traditional Chinese and Western medicine treatment has gradually emerged as a promising approach in the field of pediatric respiratory diseases. By combining the advantages of traditional Chinese medicine’s holistic regulation with Western medicine’s precise intervention, this approach has opened up new avenues for disease treatment. However, existing research has mostly focused on the combination of Chinese herbal decoctions and Western medicine, with little exploration of the application of press needle therapy, a Chinese medicine external treatment method, in the treatment of respiratory tract infections in children. As a new type of intradermal needle therapy, press needle therapy have shown great potential in the treatment of pediatric diseases due to their ease of use, low pain, long needle retention time, high safety, and long-lasting stimulation, becoming the choice of more and more parents.**^[10–13]^**

This study used a retrospective cohort study to observe the efficacy of press needle therapy combined with Western medicine in treating ALRTIs caused by RSV in children under 5 years of age, including improvements in symptoms, signs, and lung function, with the aim of providing a reference for clinical application and leveraging the unique advantages of traditional Chinese medicine.

## 2. Methods

### 2.1. General information

A total of 83 children who were hospitalized in the pediatrics department of the First People’s Hospital of Jintang County from September 2024 to April 2025 and met the diagnostic criteria for RSV lower respiratory tract infections (according to the ‘Guidelines for the Diagnosis, Treatment and Prevention of RSV Infections in Children in China (Doctor’s Edition of 2024)^[9]^) were retrospectively selected and divided into an experimental group (42 patients) and a control group (41 patients), in which the experimental group was treated using press needle therapy in combination with Western medical treatment, and the control group only used Western medical treatment. Inclusion criteria: age ≤ 5 years, hospitalization duration ≥ 5 days, complete medical records (including symptom and physical examination records, treatment details, pulmonary function test results, and discharge summary), and a standardized Western medical treatment protocol^[9]^; exclusion criteria: age >5 years, hospitalization duration < 5 days, concomitant bacterial infection/mycoplasma infection/adenovirus infection, congenital heart disease, immunodeficiency disorders, or voluntary discharge. There was no statistically significant difference between the 2 groups of children in terms of gender, age, duration of disease, condition and other general information (*P* >.05).

### 2.2. Specific methods

The experimental group of children using press needle therapy combined with western medical treatment, press needle treatment specific methods: brand: Li Yi press needle, specifications: 0.18 * 0.3 mm (under 1 year old), 0.18 * 0.5 mm (1–5 years old), acupuncture points: double lung acupoints, double fixed wheezing, retention method: each time to stay in the needle for 48 hours, 3 times a day to press, each time for 5 minutes, the course of treatment 2 to 3 times.

Western medical treatment: oral medication (Ambroxol oral liquid, Compound Pseudoephedrine Hydrochloride and Chlorpheniramine Maleate Syrup), nebulization therapy (inhaled glucocorticoids, bronchodilators, cholinergic receptor antagonists), infusion therapy (methylprednisolone 1mg/kg/dose ivgtt bid, vitamin C 0.3 g ivgtt qd).

Children in the control group only use western medical treatment, western medical treatment: oral drugs (Ambroterol oral solution, Compound Pseudoephedrine Hydrochloride and Chlorpheniramine Maleate Syrup), nebulization treatment (inhaled glucocorticosteroids, bronchodilators, cholinergic receptor antagonists), infusion therapy (methylprednisolone 1mg/kg/time ivgtt bid, vitamin C 0.3 g ivgtt qd).

### 2.3. Observation indexes

Clinical symptoms: analyse the improvement in symptoms (wheezing, coughing, shortness of breath) and signs (dry and wet rales in the lungs) after treatment in the 2 groups of pediatric patients, and compare the differences between the 2 groups.

Airway function: analyse the VT/kg (kg tidal volume), TPTEF/TE (%) (peak time ratio), VPEF/VE (%) (peak volume ratio) of the children in the 2 groups after treatment, and compare the differences between the 2 groups.

### 2.4. Statistical methods

Statistical analysis was performed using IBM SPSS Statistics 26.0 software. All quantitative data (time to resolution of clinical symptoms and signs, pulmonary function indicators) are presented as mean ± standard deviation (Mean ± SD). Data were confirmed to follow a normal distribution using the Shapiro–Wilk test (*P* > .10) and to have equal variances between groups using the Levene test (*P* > .05). Intergroup comparisons were performed using the independent samples t-test. A *P*-value <.05 was considered statistically significant.To comprehensively assess the study results, we also calculated effect sizes (Cohen d) and 95% confidence intervals. Additionally, considering potential confounding factors (such as age, baseline severity, comorbidities, etc), we conducted multivariate regression analysis to further validate the robustness of the results.

## 3. Results

Clinical symptoms: the time to relieve wheezing after treatment in the experimental group was 2.43 ± 0.51 days, and 2.86 ± 0.46 days in the control group, *P*<.01; the time to relieve shortness of breath in the experimental group was 2.46 ± 0.46 days, and 2.88 ± 0.5 days in the control group, *P*<.01; the time to relieve cough in the experimental group was 5.41 ± 0.58 days, and 5.84 ± 0.70 days in the control group. 5.84 ± 0.70 days, *P*<.05; the time of disappearance of lung signs of children in the experimental group was 5.46 ± 0.52 days, and 5.89 ± 0.52 days in the control group, *P*<.01. The difference was statistically significant, see Table 1.

**Table 1:** Comparison of changes in symptoms and signs after treatment between the 2 groups of children (d).

Group	Number of cases	Relief of wheezing	Relief of shortness of breath	Relief of cough	Relief of lung signs disappeared
Experimental group	42	2.43 ± 0.51*	2.46 ± 0.46*	5.41 ± 0.58**	5.46 ± 0.52*
Control group	41	2.86 ± 0.46	2.88 ± 0.5	5.84 ± 0.70	5.89 ± 0.52
*t*-value		3.233	2.843	2.454	3.028
*P*-value		<.01	<.01	<.05	<.01

The largest number following the ± sign is marked with "**," while the smallest number is marked with "*."

The effect size (Cohen’s d) for the time to relieve wheezing after treatment in the experimental group was 0.92, with a 95% confidence interval of (0.65–1.19); the effect size (Cohen’s d) for the time to relieve shortness of breath was 0.89, with a 95% confidence interval of (0.62–1.16); The effect size (Cohen d) for the time to relieve cough was 0.63, with a 95% confidence interval of (0.35–0.91); the effect size (Cohen d) for the time of disappearance of lung signs was 0.88, with a 95% confidence interval of (0.61–1.15).

Airway function: the VT/kg of children receiving treatment in the experimental group was 8.43 ± 1.37, compared with 7.43 ± 1.50 in the control group, *P* < .05; TPTEF/TE (%) was 28.14 ± 4.16, compared with 24.86 ± 3.09 in the control group, *P* < .01; VPEF/VE (%) was 28.21 ± 4.36, compared with VPEF/VE (%) was 28.21 ± 4.36, and that of the control group was 25.21 ± 3.16, *P*<.01. The difference was statistically significant, see Table 2.

**Table 2:** Comparison of changes in lung function after treatment in 2 groups of children.

Group	Number of cases	VT/kg	TPTEF/TE (%)	VPEF/VE (%)
Experimental group	42	8.43 ± 1.37*	28.14 ± 4.16**	28.21 ± 4.36**
*t*-control group	41	7.43 ± 1.50	24.86 ± 3.09	25.21 ± 3.16
* t*-value		2.556	3.293	2.896
* P*-value		<.05	<.01	<.01

The largest number following the ± sign is marked with "**," while the smallest number is marked with "*."

The effect size (Cohen’s d) of VT/kg in the experimental group after treatment was 0.70, with a 95% confidence interval of (0.42–0.98); The effect size (Cohen d) for TPTEF/TE (%) was 1.12, with a 95% confidence interval of (0.84–1.40); The effect size (Cohen’s d) for VPEF/VE (%) was 1.02, with a 95% confidence interval of (0.76–1.28).

Effect size, confidence intervals, and multivariate regression analysis results, see Table 3.

**Table 3:** Effect size, confidence intervals, and multivariate regression analysis results.

Indicator	Experimental group mean ± SD	Control group mean ± SD	*P*-value	Effect size (Cohen d)	95% confidence interval	Multivariate regression analysis (adjusted *P*-value)
Relief of wheezing (d)	2.43 ± 0.51	2.86 ± 0.46	<.01	0.92	(0.65–1.19)	<.01
Relief of shortness of breath (d)	2.46 ± 0.46	2.88 ± 0.50	<.01	0.89	(0.62–1.16)	<.01
Relief of cough (d)	5.41 ± 0.58	5.84 ± 0.70	<.05	0.63	(0.35–0.91)	<.05
Relief of lung signs disappeared (d)	5.46 ± 0.52	5.89 ± 0.52	<.01	0.88	(0.61–1.15)	<.01
VT/kg (mL/kg)	8.43 ± 1.37	7.43 ± 1.50	<.05	0.70	(0.42–0.98)	<.05
TPTEF/TE (%)	28.14 ± 4.16	24.86 ± 3.09	<.01	1.12	(0.84–1.40)	<.01
VPEF/VE (%)	28.21 ± 4.36	25.21 ± 3.16	<.01	1.02	(0.76–1.28)	<.01

TPTEF/TE (%) = time to peak ratio, VPEF/VE (%) = volume to peak ratio, VT/kg = kg tidal volume.

## 4. Discussion

This study used retrospective cohort analysis to confirm that the combination of press needle therapy and Western medicine has significant advantages in improving the symptoms and signs of respiratory tract infections and lung function in children. The results are closely related to traditional Chinese medicine theory and modern medical mechanisms. From the perspective of traditional Chinese medicine, respiratory tract infections in children are mostly classified as “colds” and ‘coughs,’ and the key to the pathogenesis lies in external pathogenic factors and lung deficiency. Based on meridian theory, press needle therapy selects relevant acupoints on the lung meridian and large intestine meridian, such as Lieque and Hegu. Lieque is a lung meridian acupoint that can promote lung function, relieve coughing and asthma; Hegu is a large intestine meridian acupoint that can dispel wind, relieve symptoms and activate the meridians. Press needle therapy continuously stimulates acupoints, stimulates meridian energy, regulates the circulation of qi and blood and the functions of the internal organs, thereby achieving the effect of strengthening the body and expelling evil, promoting lung function and relieving coughing.^[14]^ Children have the physiological characteristics of “delicate organs and insufficient qi and blood,” and are considered to have a “young yin and yang” or “pure yang” constitution. They have thin skin, tender flesh, clear and delicate organ qi, and are prone to recovery. They are highly sensitive to meridian and acupoint stimulation. The Ling Shu · Reverse and Obedient Fat and Thin states, “Infants have fragile flesh, little blood, and weak qi. When needling them, use a fine needle to prick shallowly and quickly pull out the needle. This can be done twice a day.” Shallow pricking can achieve the purpose of treating the disease. Therefore, press needle therapy, which is characterized by shallow pricking, has unique advantages in pediatric clinical practice.^[15]^In terms of modern medical mechanisms, press needle therapy can regulate the neuroendocrine-immune network by stimulating acupoints.^[16,17]^ Due to the rich distribution of nerve endings at acupoints, press needle stimulation can activate nerve pathways, promote neurotransmitter release, and thereby regulate the activity of immune cells and balance the levels of inflammatory factors.^[18–20]^ This process helps to achieve anti-inflammatory, analgesic, and immune function regulation, thereby exerting its therapeutic effects.

Western medical treatment, on the other hand, can directly target pathogens and respiratory symptoms, with the 2 approaches forming a complementary synergistic effect. Previous studies have shown that combining traditional Chinese medicine external treatment with Western medicine to treat respiratory diseases in children can improve clinical efficacy and shorten the course of the disease.^[21–24]^ This study further refined the specific application of press needle therapy and provided evidence of greater clinical reference value through a retrospective cohort study. However, most studies currently focus on single therapies or simple combinations of Chinese and Western medicine, with relatively few systematic studies on the combination of press needle therapy and Western medicine in the treatment of respiratory tract infections in children in terms of improving lung function. This study fills some of the gaps in this field.

The combination of press needle therapy and Western medicine has unique advantages in the treatment of respiratory tract infections in children. As a long-acting acupoint stimulation therapy, press needle therapy is easy to operate, causes little trauma to children, and has high compliance, making it particularly suitable for children who are afraid of needles and have difficulty taking medicine. Combined with Western medicine, it can not only quickly relieve acute symptoms, but also regulate the overall function of the body through pressing needles, achieving the effect of treating both the symptoms and the root cause, which is of great significance for promoting the recovery of children and reducing the recurrence of diseases.^[25,26]^

However, this study also has certain limitations. First, as a retrospective cohort study, there is a possibility of selection bias and information bias. Second, without a more in-depth subgroup analysis of factors such as acupoint selection, stimulation time, and frequency of pressing needles, it is difficult to determine the optimal treatment plan. Finally, the current study is limited to efficacy and symptom observation, lacking support from laboratory data and biochemical research indicators.

Based on the above shortcomings, prospective, multicentre, randomized controlled studies can be conducted in the future to further verify the efficacy of pressing needles combined with Western medicine in the treatment of respiratory tract infections in children and to optimize the treatment plan. At the same time, further in-depth research on the specific molecular mechanisms of pressing needles to regulate the body’s immunity and lung function will provide a more solid theoretical basis for this therapy and promote its widespread application in the treatment of respiratory diseases in children.

## 5. Conclusion

Press needle therapy combined with western medical treatment can improve the signs and symptoms and airway function of RSV acute lower respiratory tract infection in children under 5 years old,providing new ideas and methods for clinical treatment.

## Acknowledgments

We would like to express our gratitude to the pediatric department of Jin Tang County First People’s Hospital for providing the clinical data.

## Author contributions

**Conceptualization:** Juan Meng, Yanglei Tang, Qingsong Wang, Danping Huang.

**Data curation:** Juan Meng, Shuqiong Xu.

**Formal analysis:** Qingsong Wang, Junru Wang, Lingling Jiang.

**Methodology:** Qingsong Wang.

**Resources:** Juan Meng, Shuqiong Xu, Yanglei Tang.

**Supervision:** Yanglei Tang, Junru Wang, Lingling Jiang.

**Validation:** Yanglei Tang, Qingsong Wang, Weijuan Shi.

**Writing – original draft:** Juan Meng.

**Writing – review & editing:** Juan Meng.

## References

[1] Li Y Wang X Brauer DM. Global, regional, and national disease burden estimates of acute lower respiratory infections caused by respiratory syncytial virus in children under 5 years of age in 2019: a systematic analysis. Lancet. 2022;399:2047–64. 35598608 10.1016/S0140-6736(22)00478-0 PMC7613574

[2] Xiaowei H Yonggang Z Song Y. Epidemiological characteristics of respiratory syncytial virus infection in hospitalised children with acute respiratory infections in a hospital in Hubei Province, China, from 2014 to 2022: a longitudinal surveillance study. JMIR Public Health Surveill. 2023;9:e43941. 36975172 10.2196/43941 PMC10176131

[3] Huang P Xiaoxia L Hebin C. Clinical characteristics and epidemiological analysis of respiratory syncytial virus infection in children in Wuhan City, Hubei Province, China, 2019. Dis Surveill. 2020;35:987–91.

[4] Xie J Lu K Jiayu Z. Epidemiological characteristics of pathogens causing acute respiratory infections in children in Guangzhou from 2018 to 2021. Chin J Infect Dis. 2023;41:137–43.

[5] Ren K Luo R Yu D. Epidemiological characteristics of respiratory syncytial virus in 2,066 hospitalised children with acute lower respiratory tract infections in Chongqing from 2013 to 2018. Chin J Contemp Pediatr. 2021;23:67–73. 10.7499/j.issn.1008-8830.2007139 PMC7818151 33476540

[6] Li Y Johnson EK Shi T. Estimated hospitalisation burden of acute lower respiratory infections caused by respiratory syncytial virus in children under 5 years of age in 58 countries in 2019: a modelling study. Lancet Respir Med. 2021;9:175–85. 32971018 10.1016/S2213-2600(20)30322-2

[7] Teoh Z Conrey S McNeal M. Disease burden of respiratory viruses in children under 2 years of age in a community-based longitudinal US birth cohort. Clin Infect Dis. 2023;77:901–9. 37157868 10.1093/cid/ciad289 PMC10838707

[8] Smith M Kubale J Kuan G. Incidence and severity of respiratory syncytial virus infection in children aged 0–14 years in a community-based prospective cohort. Open Forum Infect Dis. 2022;9:ofac598. 36447616 10.1093/ofid/ofac598 PMC9697591

[9] Chinese Medical Education Association Paediatrics Professional Committee, Chinese Medical Association Paediatrics Branch Respiratory Subgroup, Chinese Physicians Association Respiratory Physicians Branch Paediatrics Respiratory Working Committee, . Chinese guidelines for the diagnosis, treatment, and prevention of respiratory syncytial virus infection in children (2024 Physician Edition). Chin J Pract Paediatr. 2024;39:723–32.

[10] Xiu WC Gang WJ Zhou Q. Factors influencing the efficacy of acupuncture treatment on different outcomes and their effects: a meta-regression analysis of randomised controlled trials of acupuncture. Chin J Med Aesthetics Plast Surg. 2024;30:260–6. 10.1007/s11655-023-3617-0 38212500

[11] Guodong W. Research on the Innovation of Cheng Dan'an Acupuncture Instruments (Doctoral dissertation). Nanjing University of Chinese Medicine; 2020.

[12] Liang F Yanhui M Hongyu L. Progress in research on the main clinical applications of pressing needles. Chin Med Guide. 2019;25:11.

[13] Chen S Suning G Mori M. Clinical practice guidelines for acupuncture treatment of allergic rhinitis. J Integr Tradit West Med. 2021;27:83–90. 10.1007/s11655-020-3161-0 32170521

[14] Jie H Chun C Yan Z. Meridian differentiation and treatment and its application in clinical acupuncture research: reflections and recommendations. J Acupunct. 2025;45:708–12. Chinese. 10.13703/j.0255-2930.20240321-k0002 40369930

[15] Chunling H Yao C Xinqiang N Xinmin H. Bibliometric analysis of the application of press needle therapy in paediatric diseases. Chin J Tradit Chin Med Inf. 2022;29:39–44.

[16] Li BH. Perspectives on acupoint identity. J Acupunct Meridian Stud. 2024;17:111–5. 39205613 10.51507/j.jams.2024.17.4.111

[17] Gomez LR Leon P. Recent advances in signal transduction and transmission in acupuncture: a review of biophysics in medical science. J Acupunct Meridian Stud. 2020;13:1–11. 31765838 10.1016/j.jams.2019.11.003

[18] Wang RQ Jia CS. Innovation and basic frame conception of modern acupuncture and moxibustion discipline theory system. Zhongguo Zhen Jiu. 2022;42:1170–6. Chinese. 37199210 10.13703/j.0255-2930.20211230-k0002

[19] Xiong J Qi W Yang H. Acupuncture treatment for cough-variant asthma: a meta-analysis. Evid Based Complement Alternat Med. 2021;2021:6694936. 33868443 10.1155/2021/6694936 PMC8034997

[20] Zhihan C. Acupuncture at the Zusanli acupoint can reduce the inflammatory response in AIA mice by regulating the arachidonic acid and pentose phosphate pathways. J Chromatogr B Anal Technol Biomed Life Sci. 2024;1247:124307. 10.1016/j.jchromb.2024.124307 39306868

[21] Zhang CX Bu MR Wu XM. The efficacy of acupuncture on cough-related symptom clusters in lung cancer patients: a randomised controlled trial. Eur J Oncol Nurs. 2024;70:102598. 38795440 10.1016/j.ejon.2024.102598

[22] Li B Quan CY Zheng YK. Acupuncture-related therapies for chronic cough: a systematic review and meta-analysis. Integr Med Res. 2025;14:101121. 39944112 10.1016/j.imr.2025.101121 PMC11815682

[23] Liu X-M Fei-xue Y Min-er H. Clinical care and efficacy of acupuncture combined with loratadine for paediatric allergic rhinitis (lung-spleen deficiency type). Public Health. 2019;17:134.

[24] Shi L Zhongyuan Q Qi Z. Clinical study on acupuncture treatment for paediatric allergic rhinitis based on network disease theory. J Integr Tradit West Paediatr. 2019;11:66–9.

[25] Tsai CL Lan CC Wu CW. Acupuncture point stimulation combined with conventional treatment for chronic obstructive pulmonary disease: a systematic review and network meta-analysis. Front Med (Lausanne). 2021;8:586900. 34150784 10.3389/fmed.2021.586900 PMC8211776

[26] Chen YM Xie XL Xiao PY. Acupuncture for asthma: a systematic review and meta-analysis protocol. Medicine (Baltim). 2020;99:e18457. 10.1097/MD.0000000000018457 PMC6946403 31895775

